# Attachment and stress regulation in socioeconomically disadvantaged children: Can public childcare compensate?

**DOI:** 10.1002/imhj.21878

**Published:** 2020-07-13

**Authors:** Tina Eckstein‐Madry, Bernhard Piskernik, Lieselotte Ahnert

**Affiliations:** ^1^ Faculty of Psychology, University of Vienna Vienna Austria

**Keywords:** child attachment toward a care provider, diurnal cortisol rhythm, out‐of‐home care, stress regulation, regulación del estrés, ritmo diurno de cortisol, afectividad hacia quien presta el cuidado, cuidado fuera de casa, régulation du stress, rythme de cortisol diurne, attachement à une personne prenant soin de l'enfant à la crèche, soin hors domicile, Stressregulierung, täglicher Cortisolrhythmus, Bindung an eine_n Erzieher_in, außerhäusliche Kinderbetreuung, ストレス制御, 日中のコルチゾールリズム, 保育者へのアタッチメ ント, 家庭外での保育, 压力调节, 皮质醇昼夜节律, 对看护者的依恋, 家庭外护理, تنظيم الإجهاد، وإيقاع الكورتيزول اليومي، والتعلق بمقدم الرعاية، والرعاية خارج المنزل

## Abstract

Children growing up in families experiencing socioeconomic disadvantage (SED) are at greater risk for deficits in attachment and stress regulation as compared to peers in families with more socioeconomic resources. The present study hypothesized that care providers in out‐of‐home care might help these children to compensate. We therefore investigated 60 children (*n* = 30 from SED, *n* = 30 matched counterparts from middle class) and assessed children's Attachment Q‐Sort (AQS) toward the mother and the primary care provider in childcare centers. Moreover, children's diurnal cortisol rhythm was measured based on 12 saliva samples taken across three days a week.

The disadvantaged children showed lower AQS scores with their mothers than their care providers. Compared to their counterparts, disadvantaged children also displayed heightened cortisol release and flatter cortisol profiles reflecting overall high hypothalamic‐pituitary‐adrenocortical activities and lower capacities to regulate stress. Most notably however, multilevel path modeling linked higher care provider AQS scores to decreasing cortisol release throughout the week.

## Resumen

Los niños que crecen en familias que experimentan desventajas socioeconómicas (SED) se encuentran bajo mayor riesgo de déficits en la afectividad y regulación del estrés, comparados con compañeros de familias con más recursos socioeconómicos. EL presente estudio presenta la hipótesis de que quienes cuidan a estos niños dentro del sistema público de guarderías pudieran ayudarles a compensar. Nosotros, por tanto, investigamos a 60 niños (n = 30 de SED, n = 30 niños de clase media pareados como contraparte) y evaluamos la afectividad de los niños (AQS) hacia las madres y quienes les proveían el cuidado primario en guarderías infantiles públicas. Es más, se midió el ritmo diurno de cortisol de los niños con base en 12 muestras de saliva tomadas a lo largo de tres días a la semana. Los niños con desventajas mostraron más bajos puntajes de AQS con sus madres que con quienes les prestaban el cuidado. Comparados con su contraparte, los niños con desventajas también mostraron un elevado nivel de emisión de cortisol y perfiles de cortisol más planos lo cual refleja en general un alto nivel de actividades HPA y más bajas capacidades para regular el estrés. Aún más notable, sin embargo, el modelo de trayectoria a múltiples niveles relacionó los más altos puntajes AQS del cuidador con una baja en la emisión de cortisol a lo largo de la semana.

## Résumé

Les enfants grandissant dans des familles faisant l'expérience d'un désavantage socioéconomique (abrégé ici DSE) sont à plus grand risque de déficits dans l'attachement et la régulation du stress comparés aux enfants grandissant dans des familles dotées de plus de ressources socioéconomiques.

Cette étude a pris pour hypothèse que les personnes s'occupant des enfants dans les crèches publiques pourraient aider ces enfants à compenser. Nous avons donc étudié 60 enfants (n = 30 de SED, n = 30 homologues issus de la classe moyenne) et évalué l'attachement (AQS) des enfants envers leur mère et envers les personnes prenant soin d'eux dans les crèches publiques. De plus le rythme de cortisol diurne a été mesuré basé sur des échantillons de salive pris au cours de trois jours par semaine. Les enfants de milieu désavantagé ont fait preuve de scores AQS plus bas avec leurs mères qu'avec les personnes prenant soin d'eux dans les crèches. Comparés à leurs homologues, les enfants désavantagés ont aussi fait preuve de sécrétion de cortisol plus élevée et des profils de cortisol plus plats reflétaient des activités HPA dans l'ensemble hautes et des capacités plus basses à réguler le stress. Plus particulièrement cependant une modélisation à plusieurs niveaux a établi un lien entre des scores AQS plus élevés des modes de soin à la crèche à des sécrétions de cortisol décroissantes tout au long de la semaine.

## Zusammenfassung

Bindung und Stressregulierung bei sozioökonomisch benachteiligten Kindern: Kann die öffentliche Kinderbetreuung kompensieren?

Kinder aus Familien, die sozioökonomisch benachteiligten sind (socioeconomic disadvantage; SED), haben im Vergleich zu Gleichaltrigen aus Familien mit mehr sozioökonomischen Ressourcen ein höheres Risiko für Defizite bezüglich Bindung und Stressregulierung. Die Hypothese der vorliegenden Studie ist, dass Erzieher_innen in der öffentlichen Kinderbetreuung diesen Kindern helfen können, dies zu kompensieren. Wir untersuchten 60 Kinder (n = 30 aus SED Familien, n = 30 gleichaltrige Kontrollproband_innen aus der Mittelklasse) und beurteilten deren Bindung zur Mutter sowie zur primären Bezugsperson in der öffentlichen Kindertagesstätte (AQS). Darüber hinaus wurde der tägliche Cortisolrhythmus der Kinder anhand von 12 Speichelproben gemessen, die an drei Tagen in der Woche entnommen wurden.

Die sozioökonomisch benachteiligten Kinder erzielten bei ihren Müttern niedrigere AQS‐Werte als bei ihren Erzieher_innen. Im Vergleich zur Kontrollgruppe wiesen die sozioökonomisch benachteiligten Kinder auch eine erhöhte Cortisolfreisetzung und flachere Cortisolprofile auf, was auf insgesamt hohe HPA‐Aktivitäten und geringere Kapazitäten zur Stressregulierung zurückgeführt werden kann. Insbesondere aber zeigte sich durch eine Multilevel‐Pfadmodellierung ein Zusammenhang zwischen höheren AQS‐Werten bei den Erzieher_innen und geringerer wöchentlicher Cortisolfreisetzung.

## 抄録

社会経済的に貧しい子どもたちのアタッチメントとストレス制御 abstract: 抄録 社会経済的に貧しい(SED)家族のもとで育っている子どもたちは、社会経済的な 資源のある家族のもので育っている子どもたちに比べ、アタッチメントとストレ ス制御に関して欠損しているリスクが高い。本研究では、公の保育現場の保育者 らがこのこどもたちを支援し、それを補償するのではないかと仮定した。そのた め、60人の子ども(SED:n = 30 、中産階級の同等の子ども:n = 30)を調査し、 母親と公の保育園の主な保育者に対する子どもの(AQS)アタッチメントについて 評価した。さらに、子どもの日中のコルチゾールリズムを週3日にわたり、12の 唾液サンプルを採取し、測定した。 社会経済的に貧しい子どもたちは、保育者より母親に対してより低いAQS値を示 した。中産階級の子どもたちと比較して、社会経済的に貧しい子どもたちはまた、 全体的に高いHPA活動とストレスを制御するための低能力を反映して、高いコル チゾール放出とより横ばいのコルチゾールプロファイルを示していた。しかし、 特に重要なことは、多層経路モデルが、週を通じてより高い保育者のAQS値がコ ルチゾール放出を減少させることに関係している事であった。

## 摘要

摘 要:与拥有更多社会经济资源的家庭中的同龄儿童相比, 处于社会经济劣势(SED)的家庭中长大的儿童, 在依恋和压力调节方面存在缺陷的风险更大。本研究假设公共看护机构的看护者们可能会帮助这些儿童弥补这方面的缺陷。基于此, 我们调查了60名儿童 (来自SED的儿童n = 30, 来自中产阶级的相匹配的同龄儿童n = 30) , 并评估 (AQS) 了每位儿童对他们的母亲以及公共看护机构中最初那位看护者的依恋程度。此外, 我们还根据每周中三天采集的12份唾液样本来测量这些儿童的皮质醇昼夜节律。结果显示, 社会经济劣势的儿童与其母亲的AQS得分低于他们与看护者们的得分。与其他相匹配的同龄儿童相比, 这些儿童的皮质醇释放量更高, 皮质醇分布曲线更平坦, 这反映出整体较高的HPA活动和较低的压力调节能力。但是, 值得注意的是, 多层次路径模型显示出, 在儿童与看护者AQS得分较高的整个星期中, 皮质醇释放量却在逐步下降。

## ملخص

التعلق وتنظيم الإجهاد عند الأطفال المحرومين اجتماعيا ً واقتصادياً: هل تقدم رعاية الأطفال العامة التعويض المطلوب؟

ملخص

الأطفال الذين ينشأون في أسر تعاني من الحرمان الاجتماعي والاقتصادي (SED) هم أكثر عرضة للعجز في التعلق وتنظيم الإجهاد مقارنة بأقرانهم في الأسر ذات الموارد الاجتماعية والاقتصادية الأكثر. افترضت هذه الدراسة أن مقدمي الرعاية في الرعاية العامة للأطفال قد يساعدون هؤلاء الأطفال على التعويض. ولذلك قمنا بالتحقيق مع 60 طفلاً (30 طفل من الفئات المحرومة اجتماعيا واقتصاديا و30 طفل من الطبقة المتوسطة) وقيمنا تعلق الأطفال(AQS) بالأم ومقدم الرعاية الأولية في مراكز رعاية الطفل العامة. وعلاوة على ذلك، تم قياس إيقاع الكورتيزول اليومي للأطفال على أساس 12 عينة من اللعاب تؤخذ عبر ثلاثة أيام في الأسبوع. وأظهر الأطفال المحرومون درجات أقل على مقياسAQS مع أمهاتهم مقارنة بمقدمي الرعاية. بالمقارنة مع نظرائهم، أظهر الأطفال المحرومون أيضًا إفراز كورتيزول متزايد وملامح الكورتيزول التملقية التي تعكس أنشطةHPA العالية بشكل عام وقدرات أقل لتنظيم الإجهاد. وأبرزها ومع ذلك، ربط نمذجة المسار متعدد المستويات أعلى مستوى الرعايةAQS عشرات لخفض الإفراج الكورتيزول على مدار الأسبوع.

Socioeconomic disadvantage (SED) is a widely recognized concern as long‐lasting adverse effects on children's health have been robustly and consistently reported over the lifespan (see Kim, Evans, Chen, Miller, & Seeman, 2018; Letourneau, Duffett‐Leger, Levac, Watson, & Young‐Morris, 2013; Seeman, Epel, Gruenewald, Karlamangla, & McEwen, 2010). Exposure to SED places children at greater risk for less optimal child development and health, and has also been associated with poor language, cognitive deficits, behavioral problems, and poor stress regulation during childhood (Blair & Raver, 2012; Evans & Kim, 2012, 2013).

SED is similar to low socioeconomic status (SES), which refers to occupation, income, and education, even though the term is conceptualized more broadly by Kim and colleagues (2018). SED additionally includes subjective perception of social position, difficult parental histories, and contextual indicators such as a sustained presence of stressors associated with ecological shortcomings, detrimental living environments, family chaos, poor neighborhoods, and community violence. One of the most commonly used SED indicators, however, is poverty, which is calculated using household income that falls below an annually adjusted poverty line.

Consequently, SED families encounter everyday challenges quite differently than families with greater socioeconomic resources. The perceptions of economic and ecological pressure certainly affect parenting behavior. Some studies have shown that SED parents may be less affectionate and sensitive in parent–child interactions, that is, they are more likely to use harsh disciplinary behaviors (e.g., Arditti, Burton, & Neeves‐Botelho, 2010; Iruka, Harden, Bingham, Esteraich, & Green, 2018), even though these parents often feel less effective and capable in disciplinary interactions with their children (e.g., Booth, Macdonald, & Youssef, 2018; Mistry, Vandewater, Huston, & McLoyd, 2002; Pereira, Negrão, Soares, & Mesman, 2015). These adverse care patterns influence the attachment formation negatively and contribute to insecure attachments (e.g., Coyl, Roggman, & Newland, 2002; Diener, Nievar, & Wright, 2003; Koehn, & Kerns, 2017). Moreover, numerous studies have also demonstrated that adverse parenting negatively affects child stress regulation (e.g., Bernard, Frost, Bennett, & Lindhiem, 2017; Saridjan et al., 2010; Sheridan, How, Araujo, Schamberg, & Nelson, 2013; Sheridan, Sarsour, Jutte, D'Esposito, & Boyce, 2012).

Recent stress research in children focuses on analyses of saliva cortisol as saliva sampling is noninvasive and less intrusive compared to alternative procedures. Cortisol is the primary hormonal product of the Hypothalamic‐Pituitary‐Adrenocortical (HPA) axis. The HPA axis releases cortisol according to a diurnal rhythm, with cortisol reaching the highest level after waking and declines across the day to the lowest level at night. To describe the dynamics of the stress response system closely linked to the HPA axis, theorists posit that neurobiologically mediated sensitivity to context as well as adaptive calibration through experience are responsible for the striking number of individual variations (e.g., Boyce & Ellis, 2005; Del Giudice, Ellis, & Shirtcliff, 2011). In naturalistic cortisol research, scholars mainly describe the overall shape of the cortisol diurnal rhythm by the morning‐to‐evening decline (slope) and the overall cortisol release per day (AUC: area under the curve). Individuals in contexts with fewer stressors are most likely to show less cortisol release (low AUC) and steeper slopes (larger drop in cortisol from morning to evening) than individuals in contexts with more stressors, who are most likely to show flatter slopes that may mature into cortisol dysregulation (Miller, Chen, & Zhou, 2007; Saxbe, 2008). Cortisol dysregulation manifests either as hyper‐ or hypocortisolism, depending on a consistently high or low cortisol release throughout the day. There is also consensus that cortisol dysregulation most likely indicates long‐term adaptations to stressful environments (see also Hunter, Minnis, & Wilson, 2011; Lupien, King, Meaney, & McEwen, 2001; Miller et al., 2007). Thus, flatter diurnal cortisol profiles may most reliably reflect the influence of early adverse life events on the early maturation of the stress system, which is harmed by long‐lasting negative interactions. The conditions under which these flatter slopes tend to occur, together with high or low cortisol release, are still being investigated (see Bernard et al., 2017). Some researchers found high cortisol release (e.g., Saridjan et al., 2010), others low cortisol release (e.g., Bernard, Butzin‐Dozier, Rittenhouse, & Dozier, 2010; Zalewski, Lengua, Thompson, & Kiff, 2016) in children at risk.

**Key Findings**

1. Children experiencing socioeconomic disadvantage (SED) displayed elevated stress in the form of heightened cortisol release (particularly on Sundays) and flatter diurnal cortisol decline (throughout the week), reflecting lower capacities to regulate stress as compared to their peers from families with more socioeconomic resources.
2. Children from SED families showed significantly higher attachment security to their providers in childcare than to their mothers.
3. Greater attachment security to the care providers was associated with better physiological stress regulation throughout the week, particularly in children from SED families.
4. The study thus provides evidence that care providers in public childcare have the potential to help children regulate and manage stress.

**Statement of relevance to the field of infant and early childhood mental health**

Young children's secure attachments to care providers in childcare centers may be particularly important for children at greater risk for deficits in attachment security to their parents and poor stress regulation.

Overall, stress research has demonstrated that steep diurnal slopes represent a dynamic stress system, which is capable of coping with the daily challenges. Furthermore, Pendry and Adam (2007) were able to relate the steeper cortisol rhythm to better maternal parenting quality, as assessed by a self‐reported warmth measure and mothers’ activities checklists to record her involvement. The hypothesis that early maternal care and attachment experiences modify the neural development of emotional and stress processing suggests that children's attachment security is a powerful vehicle to regulate daily challenges most effectively (Francis, Champagne, Liu, & Meaney, 1999; Perry, Blair, & Sullivan, 2017; Schore, 2001).

The fact that the HPA axis is particularly vulnerable to social stress also appears critical for children who are cared for in childcare centers. Numerous studies have demonstrated that the quality of out‐of‐home care is closely linked to children's diurnal cortisol rhythms. Children who attend low‐quality programs are more likely to demonstrate higher cortisol levels (e.g., Dettling, Parker, Lane, Sebanc, & Gunnar, 2000; Legendre, 2003; Tout, de Haan, Campbell, & Gunnar, 1998) as compared to cortisol releases of children in high‐quality centers (e.g., Ahnert, Gunnar, Lamb, & Barthel, 2004).

The present study thus hypothesized that high‐quality childcare might counteract the poor stress regulation of children who lack sensitive caregiving at home and hardly form secure attachment relationships with their parents. In more detail, children's attachments to adults outside of the family, as is typically developed in childcare, might be enriching and compensate for the adverse parent–child interaction patterns and attachment insecurity with parents. Research has shown that enrollment in childcare allows children to additionally form significant attachment relationships which help to comfort them in times of distress (for an overview see Lamb & Ahnert, 2006; Ahnert, Pinquart, & Lamb, 2006; Howes & Spieker, 2008).

As with parents, the security of care provider–child attachment, however, relates to sensitivity, involvement, and quality of care provided by care providers. Because care provider–child attachments can emerge almost independently from mother–child attachments (see meta‐analysis by Ahnert et al., 2006), care providers bear the potential to form close relationships with the children, thereby positively affecting the child's emotional balance and HPA axis activities (e.g., Badanes, Dmitrieva, & Watamura, 2012; Shields et al., 2001). On the other hand, however, children from SED families might have difficulties in establishing secure attachments, in general. These children might not have developed adequate social skills in order to easily adjust to an unknown caregiver, and care providers in childcare centers are not always able to take children's individual needs into account within a group setting (e.g., Eckstein‐Madry & Ahnert, 2016; Phillips, Voran, Kisker, Howes, & Whitebook, 1994; Ritchie & Howes, 2003). For this reason, it remains unanswered to what extent secure care provider–child attachments can develop in children from SED families. However, if they do, no study so far has answered the question of whether care providers can compensate for children's insecure attachment experiences from home.

The present study first focused on attachments of children from SED families toward their mothers as well as their childcare providers. We investigated children's attachment security at home and in the centers, and took up starting analyses that showed children's adverse behaviors can make it difficult to form secure attachments in the centers (Eckstein‐Madry & Ahnert, 2016). Second, we explored the diurnal cortisol rhythms of the children from SED families and compared them with those from middle‐class families. Finally, we examined the interplay of mother–child and care provider–child attachments in relation to children's stress regulation, aiming to discover compensatory mechanisms positively related to better child stress functioning through better attachment experiences in childcare. We hypothesized that (a) children from SED families are more likely to develop insecure attachments toward their mothers, than their counterparts from families with more socioeconomic resources. We also anticipated that (b) children from SED families might exhibit stress dysregulation indexed by higher cortisol release and flatter morning‐to‐evening declines in cortisol throughout the day. Given the professional attitudes and high standards of quality in German childcare centers (see Koenig, Leu & Viernickel, 2015), we furthermore expected (c) that care providers form secure attachments to the children (regardless of their family backgrounds), and that these care provider–child attachments (d) would buffer the insecure attachments toward the mothers, in particular in children from SED, as well as (e) contribute positively to children's stress regulation during the times in childcare, indicated by lower cortisol release and steeper slopes.

## 1 METHOD

### 1.1 Samples

The study involved *N* = 60 children (*n* = 32 girls) who were *M* = 47.4 (*SD* = 14.0) months old with their families in Saxony‐Anhalt, Germany. Of this sample, *n* = 30 children came from socioeconomically disadvantaged (SED) families, and *n* = 30 counterparts from middle‐class families with more socioeconomic resources served as comparisons.

#### 1.1.1 Recruitment and matching procedure

The Child Protection Service (CPS) helped to recruit the SED families by distributing flyers at the service centers. Different to other European countries (see a discussion on the CPS in Germany and the UK by Simpson & Nowacki, 2017), German CPS acts foremost as a preventative institution that monitors children experiencing poverty after neighborhoods, childcare institutions, or even the families themselves have called for help. The offers provided by CPS are initially relatively mild, such as sending a social worker to the home to provide in‐home services to them, enrolling the children in childcare, taking the parents to courses to improve parenting or the intrafamilial relationships, and many other activities. In the present research, CPS handed out the study information to these parents with the prospect of remuneration as compensation for the time (in hours) spent in the study (compensation was paid in Euro and negotiated after attendance). Thereby, we were able to involve hard‐to‐reach families but compensated the matched families, as well. Although CPS did not disclose the files of the families for the present study, to our knowledge, the children had faced family chaos and mild physical but not sexual abuse or other severe kinds of maltreatment.

If the families showed an interest in the study and provided written consent, we visited them at home and inquired about their SED indicators, such as SES, unemployment, incomplete occupational qualification, status of single parent, and maternal age at delivery of the first child (e.g., Erickson, Sroufe, & Egeland, 1985; NICHD, 2005). If the SED families decided to stay in the study, we searched for comparable children in the same childcare centers in which the disadvantaged children were cared for, and distributed flyers for the middle‐class families with more socioeconomic resources. In order to maximize the comparability of the two groups, middle‐class children were carefully selected regarding gender, age, and mother's age to be individually matched with the disadvantaged target children on a case by case basis. As a result, the groups did not differ regarding children's gender (53.3% girls) and age [disadvantaged: *M* = 47.9 months, *SD* = 14.4; middle‐class: *M* = 46.6 months, *SD* = 14.2; *t*(58) = 0.36, *ns*]. Furthermore, mothers’ average age was the same across the groups [disadvantaged: *M* = 27.9 years, *SD* = 7.3; middle‐class: *M* = 30.3 years, *SD* = 5.0; *t*(58) = 1.5, *ns*].

#### 1.1.2 Sample characteristics

Apart from the matched characteristics, SED families tremendously differed from the middle‐class families, particularly in terms of household income. Whereas the middle‐class families relied on an above average household income (around 13,700 Euro for single earner and 28,700 Euro for dual earner families per year), all SED families remained below the poverty line, that is, 60% of the average household income (see EU‐SILC, 2018), and were forced to rely on welfare. Furthermore, middle‐class mothers had one to three children, *M* = 1.6 (*SD* = 0.6) and 17.2% of them were 20 years and younger at first delivery. In contrast, SED mothers had one to five children (*M* = 2.2, *SD* = 1.3), and were three times more likely to be under 20 years old at the birth of their first child (46.7 % vs. 17.2 %), *χ* ^2^(1) = 6.2, *p* < .05. Additionally, SED mothers were three times more likely to be single mothers (60 vs. 20%), *χ* ^2^(1) = 10.0, *p* < .001, four times more likely to have incomplete occupational qualification (76.7 vs. 17.2%), *χ* ^2^(1) = 20.9, *p* < .001, and seven times more likely to be unemployed (73.3 vs. 10.3%), *χ* ^2^(1) = 24.0, *p* < .001.

##### 1.1.2.1 Childcare centers and care providers

In Germany, children from age one are entitled by law to attend child care for at least 20 hours per week, regardless of whether the mothers are in the labor force or not. Centers are usually open from Monday to Friday from 6:00 a.m. to 5:00 p.m (or 17:00). The children were enrolled in 13 childcare centers, which were located in poor areas in Saxony‐Anhalt (Germany). The target children attended the centers throughout the week (for at least 4 hours per day) in group settings of 11–34 children with no difference in mean sizes between the two groups, *M* = 19.8 (*SD* = 6.4) vs. *M* = 18.2 (*SD* = 2.7), *t*(56) = 1.3, *ns*. The children were cared for by 52 professional care providers (i.e., seven care providers cared for more than one child). All care providers were the primary care providers out of a team of two to three care providers per group. The primary care providers had taken care of the target children for Mdn = 18.5 months, which is a sufficient period to form and stabilize an attachment relationship (see Ereky‐Stevens, Funder, Katschnig, Malmberg, & Datler, 2018). This time did not differ between the children from SED families and those from the middle‐class, *U* = 122.0., *ns*. All primary care providers grew up in middle‐class families, had completed professional training, and acquired professional experience over a period of 6–41 years, not differing between the disadvantaged and the middle‐class group, *M* = 28.7 years (*SD* = 9.1) vs. *M* = 25.6 years (*SD* = 6.0), *t*(58) = 1.6, *ns*. Care providers of the disadvantaged as opposed to the middle‐class children, however, were significantly older, *M* = 49.2 (*SD* = 7.8) vs. *M* = 45.0 (*SD* = 6.2), *t*(58) = 2.3, *p* < .05.

### 1.2 Overall Design

The study conformed to the ethical guidelines of the German Research Foundation, which conforms with the APA rules. Care providers and parents gave informed consent prior to data collection. All families were visited at home to gather information about SED indicators. Later, we observed the mother–child attachment for at least two hours to complete the Attachment Q sort (AQS) (see in Section 1.3.1). Almost two weeks later, an observer assessed the children's attachments in the childcare centers, after identifying the primary care providers based on the longest period they had cared for the target child. We also interviewed the care providers (on personal information, family background, and career pathways) and registered information about the group settings in which the target children were enrolled.

Most importantly, saliva from the children was collected four times a day (in the morning, at noon, in the afternoon, and evening) on three days (Sunday, Monday, and Friday) of the same week to assess cortisol levels. These repeated assessments made it possible to investigate children's stress regulation under the influence of only their home (on Sunday), and with influences of childcare (on Monday and Friday).

### 1.3 Measurements

#### 1.3.1 Attachment

We assessed children's attachments with the German version of the AQS during two‐hour observations (AQS: Waters, 1995; see also Ahnert et al., 2012). The AQS captures children's attachments in their everyday environments, thus allowing for an ecological examination of attachments going beyond infancy and covering the preschool years. Resulting AQS scores range from −1 to +1 but were z‐transformed using Fisher's *r*‐to‐*z* (see Teti, Nakagawa, Das, & Wirth, 1991) in order to ensure normal distribution. In preparation for the observations, six research interns were intensively trained for the AQS procedure using video training and live observations. Applying the standard requirement, ten certification tapes determined whether the observers had reached an interrater reliability of at least ICC = .75 before they independently observed the children. However, about 10% of the observations were simultaneously carried out by two observers at the beginning of the study to ensure the reliability (which these observers confirmed with an excellent reliability of *ICC* = .90). Furthermore, we informed observers that the study included families of a broad range of SES, and randomly assigned them to the children (but no observer saw the same child at home and in the childcare center). Thus, the observers were blind to the disadvantaged status of the children as well as to the study's hypotheses. Overall, AQS scores across the two subsamples ranged from −.62 to .76 (*M* = .24, *SD* = .33) for mothers, and from −.11 to .72 (*M* = .29, *SD* = .16) for care providers.

#### 1.3.2 Child stress regulation

Each target child provided four saliva samples per day in order to describe the diurnal cortisol rhythm on a Sunday, Monday, and Friday of the same week. Sampling times were set up (a) in the morning, *M* = 8:06 a.m. (*SD* = 0:29), (b) at noon, *M* = 11:02 a.m. (*SD* = 0:21), (c) in the afternoon, *M* = 2:24 p.m. (*SD* = 0:20), and (d) in the evening, *M* = 6:12 p.m. (*SD* = 0:27).

The first author and a group of research assistants collected the saliva samples. If research assistants were unavailable, parents and care providers received a sampling kit including the saliva sampling materials, along with written and visual instructions. We instructed parents and care providers on how to collect, record, and store the saliva samples until a research assistant collected them. Substantial efforts were made to explain why exact timing and no eating or drinking shortly before sampling were essential to our study, and participants were asked to note any sampling issues that had occurred. We also conducted visits to the families (if they agreed) to help ensure compliance during the saliva samplings, particularly on Sundays. Research assistants collected 40.8%, care providers 30.5%, and parents 27.2% of the samples; subsequent cortisol measures did not differ by collectors but by time of collection as expected (see below).

No oral stimuli were used, and the saliva samples were kept in salivettes, frozen at 18°F, and stored until they were assayed. The samples were sent altogether to the Kirschbaum laboratory at the Technical University of Dresden, Germany. Using an Enzyme Immuno Assay (EIA: SynELISA Sensitive) with a sensitivity of 0.02 μg/dL in concentrations of 010 μg/dL, the intra‐ and interassay reliability for 10 μl saliva ranged from 7 to 10% in cortisol concentrations of 0.4 to 0.7 μg/dL. To minimize further variability, all samples were analyzed as duplicates and averaged. Only 1.5% of the saliva samples could not be analyzed due to the limited amount of saliva collected.

### 1.4 Data Analysis

The small sample size and the nested nature of the data provided several challenges, meaning multilevel regressions were mainly used. To examine the children's AQS scores with both the mothers and care providers in the disadvantaged and the middle‐class group, multilevel regressions accounted for the fact that some children were cared for by the same care provider. Furthermore, multilevel regressions properly handled the more complex data structure of the children's cortisol release throughout the day and week, including missing data, and also accounted for the nested structure of the data. In order to gain the most from the cortisol data, all individual cortisol levels (four cortisol levels for each of the three cortisol profiles per child) were analyzed. In preparation for these regressions, we logarithmized the raw cortisol data to obtain normal distributions and used *R* ^2^ to estimate the explained variance as suggested by Nakagawa and Schielzeth (2013). Finally, multilevel path models combined the attachment and cortisol release in order to demonstrate how the children's attachments are linked to the diurnal cortisol profiles. To reduce complexity, these models were based on the aggregated cortisol indices that describe children's stress regulation through the AUCs and the slopes of the individual diurnal cortisol rhythm. That is, AUC was accounted for, as suggested by Pruessner, Kirschbaum, Meinlschmid, and Hellhammer (2003), and the slopes were retrieved from the previous regressions. AUCs, slopes, and AQS scores were standardized before multilevel path modelling.

## 2 RESULTS

### 2.1 Attachments toward Mothers and Care Providers in Child Care Centers

**FIGURE 1:**
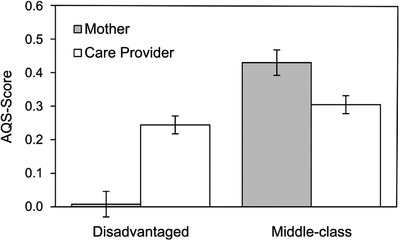
Attachment scores toward the mother and the care provider in disadvantaged and middle‐class children

We subjected all AQS scores to a multilevel regression, controlling for the nesting of the scores caused by some care providers who cared for more than one child in the group. *Child* (middle‐class vs. disadvantaged) and *Adult* (mother vs. care provider) and their interaction all significantly explained variance of *R* ^2^ = .41 of the AQS scores. Both *Child* (*b* = −0.46, *SE* = 0.05, *β_y_* = −1.76, *p* < .001) and *Adult* (*b* = −0.15, *SE* = 0.05, *β_y_* = −0.59, *p* = .003) were significantly linked to AQS scores, showing lower scores overall in disadvantaged compared to middle‐class children and lower scores overall toward the care providers than mothers. However, a *Child* × *Adult* interaction (*b* = 0.39, *SE* = 0.07, *β_y_* = −1.53, *p* < .001) was significant too, displaying opposite effects on the AQS scores: disadvantaged children scored higher in care provider–child attachments than in mother–child attachments, and the middle‐class children scored lower in care provider–child attachments than in mother–child attachments. Pairwise contrast tests (see Table 1 and Figure 1) revealed that disadvantaged children displayed no differences in the care provider AQS scores when compared to their middle‐class counterparts (0.06 *SD*), but lower AQS scores toward the mothers (to 0.46 *SD*).

**TABLE 1:** AQS scores (estimated cell means) in disadvantaged and middle‐class children

	Middle‐class	Disadvantaged	*t* (df)
Mother	.47	.02	8.8 (109.9)
Care Provider	.32	.26	1.1 (109.9)
*t* (df)	3.1 (84.3)**	5.0 (84.3)***	

*Note*. Standard error of all mean cells is *SE* = 0.04.

### 2.2 Cortisol Release throughout the Week

Testing the effect of *Child* (middle‐class vs. disadvantaged) and *Weekday* (Monday vs. Friday with Sunday as reference) on the AUCs of the diurnal cortisol profiles, a multilevel regression explained *R* ^2^ = .44 of the variance even though the nested dataset once again controlled for the multiple care structure in child care. Only the main effect of *Child* emerged significantly. This indicated that the AUCs of disadvantaged children were almost one *SD* higher (*b* = 5.97, *SE* = 1.14, *β_y_* = 1.18, *p* < .001) than the AUCs of their counterparts, confirming higher cortisol release in disadvantaged children throughout the week.

### 2.3 Cortisol Levels and Slopes throughout the Week

**FIGURE 2:**
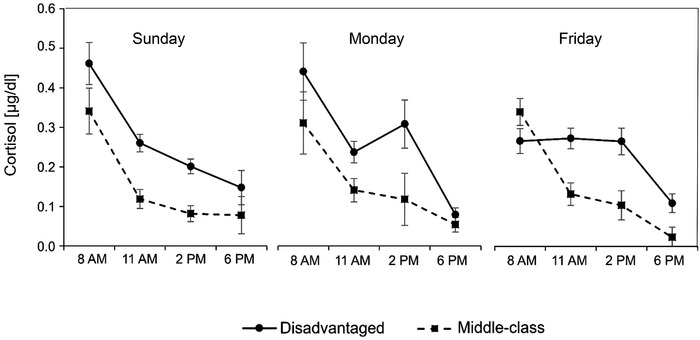
Diurnal cortisol slopes in disadvantaged and middle‐class children (Means and *SE*)

A multilevel regression also analyzed all individual cortisol levels in *Child* (middle‐class vs. disadvantaged), *Weekday* (Monday vs. Friday with Sunday as reference), and *Time* (exact times at which the cortisol levels were assessed), representing the morning‐to‐evening decline of the cortisol levels (i.e., the slopes). The regression controlled for the multiple care structure and explained *R* ^2^ = .55 of the variance. As expected for a decline of the cortisol levels throughout the day, *Time* negatively affected cortisol levels (*b* = −0.20, SE = 0.02, *β_y_* = −0.19, *p* < .001). Also, *Time* × *Child* had a significant effect on the cortisol levels (*b* = 0.06, *SE* = 0.03, *β_y_* = 0.06, *p* < .05), reflecting flatter cortisol slopes in disadvantaged than the middle‐class children; see Figure 2.

### 2.4 Children's Stress Regulation throughout the Week as Related to Attachment Experience

To analyze the complex interplay of the children's attachment experiences and diurnal cortisol rhythms, we based the next analyses on previous results which showed that disadvantaged children (i) seemed to develop insecure attachments toward their mothers but were more securely attached to their care providers, where the AQS scores were similar to the middle‐class children, and (ii) demonstrated greater stress responses (had higher cortisol release [AUCs] and flatter cortisol profiles) than their counterparts. We thus aimed to test whether the secure attachments toward the care providers might have helped the stressed children to support their diurnal cortisol rhythm.

For that reason, we subjected all stress indices (AUCs as well as slopes) to two competing multilevel path models that we later compared. The data were analyzed on two levels in each model: L2 represented the individual children and tested differences in the stress indices across *Child* (middle‐class vs. disadvantaged) and *Adult* (attachments toward the mother vs. the care provider), and L1 assessed the days of the week. Model 1 tested the effects of day of the week on the AUCs and slopes consistently for all children. In Model 2, we claim that the effects of care provider–child attachments are apparent on the Monday and Friday cortisol levels only (not on Sunday). Thus, Monday and Friday were changed to be random effects, and their variances were linked to the care provider AQS scores (as a cross‐level effect between L1 and L2), constituting interaction between the care provider AQS score and the day of the week.

After computing Model 1 based on children's AUCs, the model fitted the data perfectly with *χ* ^2^(1) = 0.00, *p* = .99, confidence interval (CFI) = 1.00, and root mean square error of approximation (RMSEA) = 0.00. For Model 2, no absolute fit indices were available due to the random effects. However, lower information criteria of Model 2 compared to Model 1, Akaike information criterion (AIC) 512.1 vs. 512.5 and sample size adjusted Bayesian information criterion (BIC) 512.6 vs. 512.9, indicated Model 2 as the better representation of the AUC data.

**FIGURE 3:**
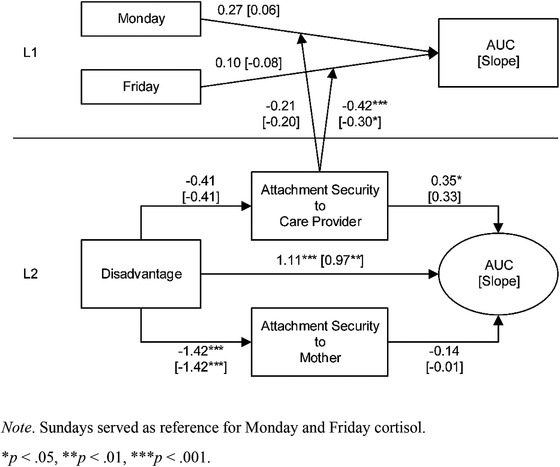
Effects of children's attachments on child stress regulation

Figure 3 displays the effect of care provider–child attachment on the AUCs (on L2 of the model) in a way that children with higher care provider AQS scores (more secure) released more cortisol (*b* = 0.35). When regarding L1 of the model, cortisol on Monday as compared to Friday did not differ when the AUC and slope was inspected. Most interestingly, however, cross‐level effects between L2 and L1 emerged when linking children's AQS scores (to their care providers) to the Monday and Friday cortisol. Whereas the Monday AUC and slope with better AQS scores were not significantly lowered *b* = −0.21 [−0.20], the Friday AUC and slope showed a significant decline with *b* = −0.42 [‐0.30]. This suggests that the cortisol release of children with higher AQS scores to their care providers significantly drops during the week until Friday (see Table 2 and Figures 2, 3).

**TABLE 2:** Parameter estimates of two multilevel path models computed for both AUCs and slopes of the children's cortisol profiles

	AUC				Slope			
	Model 1		Model 2		Model 1		Model 2	
	*b*	*p*	*b*	*p*	*b*	*p*	*b*	*p*
Level 1								
Monday	.19	.26	.27	.13	−.02	.92	.06	.71
Friday	−.06	.72	.10	.56	−.19	.25	−.08	.66
Level 2								
Disadvantage to								
CP Attachment	−.41	.16	−.41	.16	−.41	.16	−.41	.16
M Attachment	−1.42	<.001	−1.42	<.001	−1.42	<.001	−1.42	<.001
AUC/Slope	1.09	.001	1.11	<.001	.97	.01	.97	.01
AUC/Slope on								
CP attachment	.14	.21	.35	.02	.16	.26	.33	.14
M attachment	−.15	.38	−.14	.37	.00	.99	−.01	.97
Cross‐Level Interaction								
CP attachment ×								
Monday	‐	‐	−.21	.30	‐	‐	−.20	.40
Friday	‐	‐	−.42	.001	‐	‐	−.30	.03

*Note*. M = mother, CP = care provider, *b* = unstandardized regression coefficient, *p* = significance level.

Sunday served as the reference for Monday and Friday cortisol

## 3 DISCUSSION

In this study, we explored the supporting function of attachment experiences with care providers in childcare centers on children's stress regulation, specifically for children from SED families, characterized by economic risk and adverse parenting patterns. These disadvantaged children had developed insecure attachments to their mothers. This confirms previous studies that revealed mother–child attachments to be harmed in SED families due to elevated parenting stress and insensitive parenting (e.g., Coyl et al., 2002; Forbes, Evans, Moran, & Pederson, 2007; Mistry et al., 2002; Puckering, 2004).

In contrast, child attachments toward the care provider in childcare centers appeared as secure in disadvantaged children as was the case in the middle‐class counterparts. This outcome is not self‐evident as the childcare providers in this study grew up in middle‐class families and were unexperienced with poverty. During the vocational training, however, they learnt to reflect and to deal with children from different social backgrounds (see Koenig et al., 2015). This might explain why the care providers were able to form secure attachments regardless of the social backgrounds of the children, assuming that longer work and life experience additionally supported this process.

In contrast, Ritchie & Howes (2003) reported mainly insecure attachments of children from poor homes toward their care providers, even if the providers were highly sensitive in the interactions with these children. The Californian childcare centers that Ritchie & Howes (2003) investigated had to care for entire groups of high‐risk children, which might have complicated the interaction and attachment processes with the providers. Individualized interactions are difficult to develop for care providers in group settings where attachments might be maintained by group‐oriented interaction strategies rather than dyadic interactions with individual children (Ahnert et al., 2006; van Schaik, Leseman, & de Haan, 2017; van Schaik, Leseman, & Huijbregts, 2014). As is generally known, care providers need to adjust their behaviors according to group dynamics, and this might be difficult if the entire group is challenging (e.g., Phillips et al., 1994).

The present study, however, investigated children from SED families as part of group settings mainly caring for middle‐class children with a maximum of two disadvantaged children per group. Thus, the small numbers of disadvantaged children per group might have helped to provide sufficient attachment‐building interactions with the care providers in the present study and made compensatory attachment experiences for the disadvantaged children possible.

Children from SED families tended to display higher cortisol release and flatter diurnal cortisol slopes, reflecting more difficulties with stress regulation than their counterparts from the middle class. These results are supported by previous studies which indicated long‐lasting activation of the stress system due to stressful life circumstances (Evans & English, 2002; Flinn, 2006; Saridjan et al., 2010), and poor maternal parenting quality (Pendry & Adam, 2007). In contrast, the secure attachment to the mother holds children's arousal in check or even helps to down‐regulate it (e.g., Ahnert et al., 2004; Gunnar & Quevedo, 2007), while impaired mother–child attachments work against effective stress regulation in children (e.g., Luijk et al., 2010).

In contrast, children's attachment experience with childcare providers in childcare centers might be a relatively new experience. The present study gives first indications that this experience might be associated with the child's stress regulation in such a way that better attachment security to a care provider can predict a decrease of the cortisol release particularly at the end of a week. Interestingly, the highest cortisol release was generally linked to secure care provider attachments, suggesting that the children who were most stressed received intense attention and care (see also Ahnert, Eckstein‐Madry, Piskernik, Porges, & Lamb, in prep). These findings hold true for both the disadvantaged children and their counterparts. However, the down‐regulation of the stress in children from SED families was the greatest benefit, as the stress levels generally appeared higher in this group than those of the counterparts.

This study must be interpreted with regard to its limitations, one of which is the small sample size, which is always problematic when analyzing complex research questions. However, the cortisol levels were collected 12 times per child and this assured the statistical power of the main results. In contrast, the AQS scores were not collected repeatedly and could have been more susceptible to sampling artifacts. Nevertheless, the attachment effects were consistent and substantial in size. We also computed statistical models describing the interplay of children's attachment and stress regulation with two cortisol indices (i.e., cortisol slopes and AUCs) to assure that the interpretation is reliable, while being robustly backed up by two similar results. Thus, we can trust the results even though they are not as easily able to be generalized and further research is warranted. Larger samples, for example, would allow the impact of family characteristics on child attachment, stress regulation, and their interplay to be explored in more detail, rather than relying on a comparison of SED‐ and CPS‐involved and middle‐class homes. By involving an additional control group of children from low SES homes where CPS is not involved, the effects of poverty and intrafamilial problems and parenting stress could be explored separately from each other. Also, more records of daily routines in childcare and at home (such as napping, eating, and sleeping times) would allow an improvement in the estimates of the children's cortisol rhythms. Finally, a follow‐up study of the sample would shed light on the long‐term effects of care provider–child attachments on children's stress regulation in childcare.

The present study might also have led to more hard‐to‐reach families participating in this study, due to the remuneration for the poor homes, while the different motivation for participation in the middle‐class families might have confounded with more secure mother–child attachments. However, results convincingly showed that disadvantaged homes influence mother–child attachment and child stress regulation negatively. The better the attachments the disadvantaged children experienced in childcare centers, the better they helped these children with their stress regulation. Because secure care provider–child attachments are crucial to the overall quality of childcare centers, the present study made clear that attending high‐quality childcare not only contributes to children's mental but clearly also to their physical health.

## CONFLICT OF INTEREST

The authors declare no conflict of interest.
